# Association of tinnitus with obstructive sleep apnea and rapid eye movement-related obstructive sleep apnea in similar hearing threshold groups

**DOI:** 10.1016/j.bjorl.2025.101661

**Published:** 2025-07-08

**Authors:** Sun Choi, Seok Hyun Park, Su Yeon Kim, Su Kyoung Park, Sang Chul Park, Jiwon Chang

**Affiliations:** a Hallym University College of Medicine, Kangnam Sacred Heart Hospital, Department of Otorhinolaryngology-Head and Neck Surgery, Seoul, Republic of Korea; b Korea University College of Medicine, Department of Otorhinolaryngology–Head & Neck Surgery, Seoul, Republic of Korea

**Keywords:** Obstructive sleep apnea, Hearing, Tinnitus, REM sleep

## Abstract

**Objectives:**

Obstructive sleep apnea, a multisystem disease, affects cochlear and vestibular functions due to chronic intermittent hypoxia and recurrent oxygen desaturation. During rapid eye movement sleep, the muscle tone of the upper airway decreases more, and the cardiovascular complication risk is higher than that during non-rapid eye movement sleep. We analyzed whether tinnitus was associated with obstructive sleep apnea and rapid eye movement-related obstructive sleep apnea in patients with similar hearing thresholds.

**Methods:**

We evaluated patients with obstructive sleep apnea without (24 patients; group 1) and with (27 patients; group 2) tinnitus. All participants underwent pure-tone audiometry, tinnitus tests, polysomnography, and sleep questionnaires.

**Results:**

The sleep efficiency (86.23% ± 10.38% vs. 81.27% ± 8.10%) and Epworth Sleepiness Scale (8.50 ± 4.31 vs. 5.15 ± 2.82) values were higher in group 2 versus group 1. In patients with rapid eye movement-related obstructive sleep apnea, the sleep efficiency value was higher in group 2 (90.55% ± 4.61%) versus group 1 (78.13% ± 11.70%). However, in patients with obstructive sleep apnea and rapid eye movement-related obstructive sleep apnea, medication use that might affect sleep was higher in group 2 (44.4% and 54.4%, respectively) versus group 1 (16.7% and 28.6%, respectively).

**Conclusions:**

We evaluated the relationship between tinnitus and obstructive sleep apnea or rapid eye movement-related obstructive sleep apnea in groups with similar hearing thresholds for the first time. The presence of tinnitus in patients with obstructive sleep apnea with similar hearing thresholds was more closely related to subjective parameters than to objective parameters. Patients with tinnitus had poorer sleep quality than those without tinnitus.

**Level of evidence:**

4.

## Highlights

• OSA patients with tinnitus had better sleep efficiency than those without tinnitus.
• OSA patients with tinnitus took more sleep medications than those without tinnitus.
• Tinnitus in OSA patients was more related to subjective than objective parameters.

## Introduction

Obstructive Sleep Apnea (OSA) is a sleep disorder characterized by repeated episodes of breathing cessation leading to partial or complete airway collapse. It is relatively common, with a prevalence of 9%–38% in the general adult population.^1^, ^2^ OSA can cause fatigue and daytime sleepiness due to decreased blood oxygen saturation or sleep quality disruption caused by arousals for breathing. OSA is a multisystem disease that can lead to various diseases of the cardiovascular, metabolic, and respiratory systems due to chronic intermittent hypoxemia and repeated oxygen desaturation.^3^, ^4^, ^5^

Sleep can be divided into two stages based on behavioral, brainwave, electromyogram, and electrooculogram criteria: non-Rapid Eye Movement (non-REM) and Rapid Eye Movement (REM) sleep. During REM sleep, the muscle tone of the upper airway decreases more than that during non-REM sleep, leading to a greater likelihood of airway closure or collapse, exacerbation of hypoxemia, and sympathetic nerve activation.^6^ REM-related OSA is diagnosed when the overall Apnea-Hypopnea Index (AHI) is ≥5, the AHI during non-REM sleep is <15, and the ratio of AHI during REM sleep to AHI during non-REM sleep is ≥2.^7^, ^8^ OSA during REM sleep increases the risk of cardiovascular complications compared to OSA during non-REM sleep.^7^, ^9^

OSA can also affect the auditory and vestibular systems because of chronic intermittent hypoxemia and recurring oxygen desaturation.^10^ Studies have reported a higher incidence of hearing impairment in patients with OSA than in those without OSA.^11^, ^12^ Additionally, hearing threshold is reported to be related to OSA, and the pure-tone hearing thresholds at 250 Hz; 2,000 Hz; 4,000 Hz and 8,000 Hz are higher in patients with OSA than in those without OSA.^11^, ^13^, ^14^

Tinnitus is the perception of sound without any external sound stimulus and has a prevalence rate of 6.6%–18.6%, with a high prevalence of 30% in older individuals.^15^ Various factors such as inner ear disease, noise exposure, trauma, medications, and hearing loss, can cause tinnitus, with hearing impairment being the most common cause^16^; however, hearing loss does not always cause tinnitus, and patients with tinnitus do not always experience hearing loss. Tinnitus is noticeable in quiet environments, and 71% of patients with tinnitus report sleep disturbances. Conversely, sleep disorders can be considered a potential underlying cause of tinnitus.^17^

Recently, an association between tinnitus and OSA has been revealed^18^, ^19^, ^20^; however, research considering hearing threshold and tinnitus together when analyzing their relationship to OSA is lacking. Therefore, this study aimed to analyze the relationship between tinnitus and OSA and sleep quality in patients with similar pure-tone hearing thresholds using Polysomnography (PSG), audiometry, and sleep-related questionnaires.

## Methods

### Participants

We retrospectively analyzed patients aged ≥19-years who visited our Department of Otolaryngology Head and Neck Surgery between October 2019 and December 2022 with an OSA diagnosis. Patients with obvious external and middle ear disorders, congenital hearing loss, a history of ototoxic drug use, head trauma, or previous ear surgery that could have caused their otologic symptoms were excluded from the study. Fifty-one patients were included in the study and divided into two groups based on the presence or absence of tinnitus. Twenty-four patients without tinnitus were included in group 1, and 27 patients with tinnitus were included in group 2. Patients in both groups had similar pure-tone hearing thresholds to exclude the influence of hearing thresholds on tinnitus. The patients’ baseline conditions, including medication history, weight, height, and Body Mass Index (BMI), were investigated.

This study was approved by the Institutional Review Board of our institution (IRB 2021-12-010) and complied with the principles embodied in the Declaration of Helsinki. Written informed consent was obtained from all participants involved in the study. All data collected for this study were deidentified using participant codes to guarantee confidentiality and anonymity. The data were accessed for research purposes from 12 January 2022 to 10 December 2022.

### Audiometric assessment

All participants underwent hearing tests using an audiometer (AUDIOSTAR PRO; Eden Prairie, MN, USA) in a Soundproof Audiology booth (SAD-2200). Pure-tone thresholds at frequencies 250 Hz, 500 Hz, 1 kHz, 2 kHz, 3 kHz, 4 kHz, 6 kHz, and 8 kHz were measured using a mixed method. Hearing thresholds of 500 Hz, 1 kHz, 2 kHz, and 3 kHz were used to calculate pure-tone averages. Additionally, we checked the worst hearing threshold at any frequency (decibel Hearing Level [dB HL]) from 250 Hz to 8 kHz where tinnitus may occur and this was compared between the two groups.

Furthermore, tinnitus assessment was conducted using the same machine as the pure-tone audiometry. After identifying the tinnitus location, pitch matching was performed on the affected side, where the patients identified the pitch closest to their perceived tinnitus frequency. Subsequently, loudness matching was conducted, where the loudness of the stimulus sound was adjusted until it matched the loudness of the patient’s tinnitus at the tinnitus frequency. Additionally, based on the pitch and loudness matching results, the minimal masking level, which represents the minimum level of external sound required to mask tinnitus, was determined. Additionally, residual inhibition tests were performed by providing sufficient sound to mask tinnitus and observing whether tinnitus temporarily diminished.

### Polysomnography assessment

A standard overnight study was conducted using a computerized PSG device (Embla, Ontario, Canada), and a level 1 examination was performed. Result interpretation was based on the American Academy of Sleep Medicine scoring manual.^21^ Apnea was defined as a minimum 10-second reduction in airflow with a ≥90% reduction, whereas hypopnea was defined as a minimum 10-second reduction in airflow with a ≥30% decrease in oxygen saturation. Sleep staging was determined by analyzing digital data that recorded the time spent in each sleep stage and the latency to sleep onset. Various parameters were measured during sleep, including wake time, sleep efficiency, AHI, Respiratory Disturbance Index (RDI), position-specific AHI (supine and lateral AHI), lowest oxygen saturation during sleep, snoring time, arousal index, and periodic leg movements.

### Survey with questionnaires

We used the Stanford Sleepiness Scale and the Epworth Sleepiness Scale (ESS) to assess daytime sleepiness to measure subjective discomfort in patients with sleep apnea. Additionally, we administered the STOP-BANG questionnaire to screen for sleep apnea (STOP-BANG stands for snoring, tiredness, observed apnea, [blood] pressure, BMI, age, neck circumference, and sex). We used the Tinnitus Handicap Inventory to measure subjective symptoms related to tinnitus. Furthermore, we evaluated insomnia using the Insomnia Severity Index (ISI) and assessed anxiety and depression using the Beck Depression Inventory-II (BDI-II) and Hospital Anxiety and Depression Scale (HADS) to minimize potential bias from other factors that could affect sleep. All the questionnaires were administered when the patients visited the hospital as part of their routine treatments.

### Statistical analysis

Statistical analyses were performed using SAS software (version 9.4 SAS Institute Inc., Cary, NC, USA). The two groups were further divided into group 1 without tinnitus and group 2 with tinnitus. Age, sex, BMI, hearing thresholds, questionnaire scores, medication usage rate, and various parameters from the sleep study were analyzed using the Mann–Whitney *U* test and Chi-Square test. Statistical significance was set at p < 0.05.

## Results

### Comparison between the two groups within the total patients

The differences in age, sex, BMI, and hearing threshold between groups 1 and 2 were statistically insignificant (Table 1). However, the proportion of patients taking medications that could affect sleep was significantly higher in group 2 (44.4%) than in group 1 (16.7%) (p = 0.017).

**Table 1:** Characteristics of total patients.

	Group 1	Group 2	p-value
Number of patients	24	27	
Age	56.50 ± 15.53	58.30 ± 10.48	0.835
Sex (Male:Female)	17:7	16:11	0.560
BMI	26.95 ± 3.78	26.80 ± 4.09	0.902
PTA (dB HL)	(R) 20.83 ± 13.00	(R) 23.26 ± 14.41	0.527
	(L) 19.83 + 12.11	(L) 24.96 ± 14.22	0.114
The worst hearing threshold at any frequency (dB HL)	56.5 dB HL (30–100 dB HL)	60.5 dB HL (35–95 dB HL)	0.724
Medications related to sleep	16.7% (4/24)	44.4% (12/27)	**0.017**^a^
Treatment of OSA			0.550
Surgery (nasal or pharynx)	41.7% (10/24)	22.2% (6/27)	0.226
Medication	16.7% (4/24)	29.6% (8/27)	0.335
PAP therapy	33.3% (8/24)	44.4% (12/27)	0.567
Surgery + PAP therapy	4.2% (1/24)	0% (0/27)	0.471
No treatment	4.2% (1/24)	3.7% (1/27)	1.000

BMI, Body Mass Index; PTA, Pure-Tone Threshold Average; dB HL, Decibel Hearing Level; R, Right; L, Left; OSA, Obstructive Sleep Apnea; PAP, Positive Airway Pressure.

a Indicates a significant difference at p < 0.05. Statistically significant values are indicated in bold in the table.

Furthermore, the proportion of the N1 sleep stage was 31.73% ± 13.00% in group 1 and 26.33% ± 13.35% in group 2. Both groups exhibited a higher proportion of the N1 sleep stages than the reference value, indicating light sleep. The proportions of the N3 sleep stage were 2.59% ± 3.35% and 3.65% ± 5.68%, in groups 1 and 2, respectively. Both values were far below the reference value of 20% that typically represents deep sleep, suggesting poor sleep quality in both groups (Table 2).

**Table 2:** Sleep architecture of total patients.

	Group 1	Group 2	p-value
Total sleep time (min)	320.00 ± 26.81	331.61 ± 28.78	**0.041**^a^
N1 (min)	147.40 ± 139.74	99.48 ± 50.67	0.072
N1 (%)	31.73 ± 13.00	26.33 ± 13.35	0.122
N2 (min)	116.60 ± 46.64	146.28 ± 47.42	**0.029**^a^
N2 (%)	30.63 ± 12.77	39.01 ± 13.27	**0.028**^a^
N3 (min)	10.31 ± 13.29	13.98 ± 21.61	0.983
N3 (%)	2.59 ± 3.35	3.65 ± 5.68	0.967
REM (min)	71.32 ± 28.21	71.88 ± 31.30	0.910
REM (%)	18.59 ± 6.94	19.14 ± 8.26	0.777
Sleep latency to N1	11.44 ± 9.69	9.40 ± 12.53	0.089
Sleep latency to N2	29.23 ± 25.98	17.54 ± 26.46	**0.024**^a^
Sleep latency to N3	48.62 ± 69.86	21.67 ± 39.55	0.287
Stage REM latency from sleep onset	128.31 ± 63.21	104.17 ± 58.83	0.107

REM, Rapid Eye Movement.

a Indicates a significant difference at p < 0.05. Statistically significant values are indicated in bold in the table.

The arousal index during sleep was 16.44% ± 7.72% in group 1 and 11.91% ± 9.34% in group 2, with group 1 showing a higher value (p = 0.016) than group 2. Sleep efficiency was 81.27% ± 8.10% in group 1 and 86.23% ± 10.38% in group 2, with group 2 having a higher efficiency (p = 0.007) than group 1. Other parameters such as AHI, positional AHI, and RDI were comparable between the two groups. Furthermore, the differences between the two groups in terms of oxygen saturation, snoring time, and heart rate were statistically insignificant. However, the percentage of REM-related OSA tended to be higher in group 2 (40.74%) than in group 1 (29.17%) (p = 0.310) (Table 3).

**Table 3:** Sleep efficiency, respiratory disturbance, and oxygen status of total patients.

	Group 1	Group 2	p-value
Wake (min)	76.27 ± 40.04	56.19 ± 48.67	**0.008**^a^
Wake (%)	16.44 ± 7.72	11.91 ± 9.34	**0.016**^a^
Sleep efficiency (%)	81.27 ± 8.10	86.23 ± 10.38	**0.007**^a^
No of awakenings	38.00 ± 18.33	34.67 ± 22.06	0.230
Arousal index	53.61 ± 22.14	55.31 ± 17.38	0.993
Apnea arousals index	13.23 ± 15.43	12.15 ± 14.17	0.651
Spontaneous arousals index	9.19 ± 7.66	8.27 ± 5.45	0.993
AHI	37.45 ± 21.69	34.84 ± 23.11	0.664
Apnea index	13.90 ± 16.24	12.64 ± 14.72	0.651
Hypopnea index	23.55 ± 14.89	22.20 ± 13.28	0.902
Supine AHI	46.83 ± 26.91	46.39 ± 27.35	0.955
Lateral AHI	33.21 ± 37.35	25.72 ± 27.09	0.595
Respiratory disturbance index	49.58 ± 18.39	46.82 ± 20.44	0.692
Lowest oxygen saturation (%)	83.29 ± 7.68	84.30 ± 5.39	0.985
Mean oxygen saturation (%)	95.03 ± 1.16	94.80 ± 1.46	0.515
Sleep time with SpO_2_ < 90% (min)	7.84 ± 10.85	8.96 ± 16.22	0.677
SpO_2_ statistics: saturation < 90% (%)	2.56 ± 3.62	2.69 ± 4.68	0.593
Oxygen desaturation index	22.44 ± 17.93	21.84 ± 19.13	0.720
Snore time (min)	189.60 ± 81.63	196.00 ± 76.66	0.985
Snore time (%)	59.20 ± 24.94	58.68 ± 21.58	0.806
Mean heart rate (bpm)	64.14 ± 7.69	65.58 ± 9.15	0.678
Periodic limb movement/h	26.99 ± 41.11	10.76 ± 18.92	0.854
REM-related OSA	29.17%	40.74%	0.310
REM AHI	45.28 ± 21.80	41.34 ± 24.87	0.497
NREM AHI	34.89 ± 23.38	31.89 ± 25.40	0.597

AHI, Apnea-Hypopnea Index; SpO_2_, Oxygen Saturation; REM, Rapid Eye Movement; OSA, Obstructive Sleep Apnea; NREM, non-REM.

a Indicates a significant difference at p < 0.05. Statistically significant values are indicated in bold in the table.

Regarding sleep-related questionnaires, the BDI-II score was higher in group 2 patients (19.08 ± 13.71) than in group 1 (11.74 ± 9.04), and this difference was statistically significant (p = 0.047) (Table 4). Additionally, the ESS score, a measure of daytime sleepiness, was higher in group 2 (8.50 ± 4.31) than in group 1 (5.14 ± 2.82), and this difference was statistically significant (p = 0.010) (Table 4).

**Table 4:** Questionnaire results of total patients.

	Group 1	Group 2	p-value
Insomnia Severity Index (0–28)	9.64 ± 5.24	11.00 ± 6.60	0.461
Beck Depression Inventory (0–63)	11.74 ± 9.04	19.08 ± 13.71	**0.047**^a^
Hospital Anxiety and Depression Scale (0–42)	12.05 ± 7.49	15.41 ± 7.87	0.072
Tinnitus Handicap Inventory (0–100)	0 ± 0	28.15 ± 25.29	**0.001**^a^
STOP-BANG (0–8)	3.68 ± 1.64	3.73 ± 1.56	0.896
Berlin questionnaire (0–10)	5.60 ± 2.97	6.08 ± 2.98	0.809
ESS (0–24)	5.14 ± 2.82	8.50 ± 4.31	**0.010**^a^
SSS (0–7)	3.95 ± 3.52	2.96 ± 1.15	0.563

STOP-BANG, Snoring, Tiredness, Observed apnea, (blood) Pressure, Body Mass Index, Age, Neck circumference, and sex; ESS, Epworth Sleepiness Scale; SSS, Stanford Sleepiness Scale.

a Indicates a significant difference at p < 0.05. Statistically significant values are indicated in bold in the table.

### Comparing the two groups within the patients with REM-related OSA

Additional comparisons were performed between patients with REM-related OSA in groups 1 and 2. The differences in age, sex, BMI, and hearing threshold between the two groups were statistically insignificant. However, the proportion of patients taking medications that could affect sleep was significantly higher in group 2 (54.4%) than in group 1 (28.6%); p = 0.043 (Table 5).

**Table 5:** Characteristics of patients with REM-related OSA.

	Group 1	Group 2	p-value
Number of patients	7	11	
Age	61.29 ± 12.58	53.00 ± 8.25	0.102
Sex (Male:Female)	3:4	5:6	0.914
BMI	26.39 ± 4.75	26.82 ± 3.01	0.593
PTA (dB HL)	(R) 23.71 ± 17.64	(R) 18.27 ± 12.00	0.485
	(L) 24.29 ± 18.66	(L) 20.73 ± 9.81	0.373
The worst hearing threshold at any frequency (dB HL)	62.1 dB HL (30–95 dB HL)	52.7 dB HL (30–70 dB HL)	0.681
Medications related to sleep	28.6% (2/7)	54.4% (6/11)	**0.043**^a^
Treatment of OSA			0.206
Surgery (nasal or pharynx)	57.1% (4/7)	18.2% (2/11)	0.141
Medication	14.3% (1/7)	54.5% (6/11)	0.151
PAP therapy	28.6% (2/7)	18.2% (2/11)	1.000
No treatment	0% (0/7)	9.1% (1/11)	1.000

REM, Rapid Eye Movement; OSA, Obstructive Sleep Apnea; BMI, Body Mass Index; PTA, Pure-Tone threshold Average; dB HL, Decibel Hearing Level; R, Right; L, Left; PAP, Positive Airway Pressure.

a Indicates a significant difference at p < 0.05. Statistically significant values are indicated in bold in the table.

Comparing the sleep stages revealed no significant differences in the proportions of the non-REM stage, REM stage, and latency between the two groups (Table 6).

**Table 6:** Sleep architecture of patients with REM-related OSA.

	Group 1	Group 2	p-value
Total sleep time (min)	315.24 ± 32.68	335.78 ± 26.25	0.160
N1 (min)	186.77 ± 252.62	74.85 ± 32.35	0.077
N1 (%)	25.31 ± 8.61	20.75 ± 9.39	0.222
N2 (min)	127.11 ± 31.13	159.14 ± 36.06	0.070
N2 (%)	32.80 ± 10.18	43.64 ± 9.94	0.063
N3 (min)	12.57 ± 16.32	21.36 ± 24.95	0.602
N3 (%)	2.99 ± 3.82	5.68 ± 6.67	0.538
REM (min)	74.50 ± 20.65	80.43 ± 30.98	0.821
REM (%)	19.24 ± 6.21	21.81 ± 7.42	0.556
Sleep latency to N1	12.83 ± 13.57	5.23 ± 3.70	0.111
Sleep latency to N2	46.69 ± 36.79	8.59 ± 4.07	**0.021**^a^
Sleep latency to N3	50.33 ± 79.10	21.32 ± 23.46	0.887
Stage REM latency from sleep onset	129.93 ± 61.29	83.68 ± 21.62	0.051

OSA, Obstructive Sleep Apnea; REM, Rapid Eye Movement.

a Indicates a significant difference at p < 0.05. Statistically significant values are indicated in bold in the table.

However, the difference in sleep efficiency was statistically significant (p = 0.006), with group 1 showing 78.13% ± 11.70% and group 2 showing 90.55% ± 4.61%. The proportion of snoring time tended to be higher in group 2 (67.42% ± 21.06%) than in group 1 (50.69% ± 22.56%) (Table 7).

**Table 7:** Sleep efficiency, respiratory disturbance, and oxygen status of patients with REM-related OSA.

	Group 1	Group 2	p-value
Wake (min)	94.13 ± 60.33	35.45 ± 18.74	**0.008**^a^
Wake (%)	19.63 ± 10.97	8.19 ± 4.08	**0.007**^a^
Sleep efficiency (%)	78.13 ± 11.70	90.55 ± 4.61	**0.006**^a^
No of awakenings	33.43 ± 22.23	25.36 ± 8.13	0.526
Arousal index	45.34 ± 22.40	46.67 ± 13.70	0.441
Apnea arousals index	7.27 ± 9.62	3.08 ± 2.88	0.469
Spontaneous arousals index	13.86 ± 9.20	9.83 ± 7.14	0.342
AHI	20.47 ± 16.11	22.12 ± 13.24	0.618
Apnea index	7.64 ± 9.72	3.25 ± 2.90	0.297
Hypopnea index	12.83 ± 7.18	18.87 ± 11.69	0.257
Supine AHI	29.70 ± 19.70	32.19 ± 25.09	0.964
Lateral AHI	22.86 ± 24.48	21.02 ± 23.68	0.555
Respiratory disturbance index	38.63 ± 15.38	35.65 ± 13.06	0.618
Lowest oxygen saturation (%)	85.43 ± 7.14	86.73 ± 3.88	0.928
Mean oxygen saturation (%)	95.37 ± 0.92	95.29 ± 1.36	0.717
Sleep time with SpO_2_ < 90% (min)	2.83 ± 6.41	1.53 ± 1.61	0.461
SpO_2_ statistics: saturation < 90% (%)	0.91 ± 2.12	0.46 ± 0.49	0.374
Oxygen desaturation index	11.30 ± 6.81	11.66 ± 7.51	0.964
Snore time (min)	159.31 ± 67.67	225.65 ± 72.25	0.077
Snore time (%)	50.69 ± 22.56	67.42 ± 21.06	0.113
Mean heart rate (bpm)	67.26 ± 8.31	64.50 ± 8.34	0.441
Periodic limb movement / h	40.83 ± 56.55	11.67 ± 27.63	0.274

REM, Rapid Eye Movement; OSA, Obstructive Sleep Apnea; AHI, Apnea-Hypopnea Index; SpO_2_, Oxygen Saturation.

a Indicates a significant difference at p < 0.05. Statistically significant values are indicated in bold in the table.

In the sleep-related questionnaires, the differences between the two groups in the ISI, BDI-II, HADS, and STOP-BANG were statistically insignificant. The BDI-II score tended to be higher in group 2 (17.67 ± 12.69) than in group 1 (11.00 ± 5.73) (p = 0.091). Moreover, the ESS score tended to be higher in group 2 (9.55 ± 4.89) than in group 1 (5.33 ± 3.44); p = 0.096 (Table 8).

**Table 8:** Questionnaire results of patients with REM-related OSA.

	Group 1	Group 2	p-value
Insomnia Severity Index (0–28)	11.57 ± 7.14	11.60 ± 5.25	0.854
Beck Depression Inventory (0–63)	11.00 ± 5.73	17.67 ± 12.69	0.091
Hospital Anxiety and Depression Scale (0–42)	10.67 ± 6.83	14.78 ± 7.95	0.158
Tinnitus Handicap Inventory (0–100)	0 ± 0	25.09 ± 19.79	**0.001**^a^
STOP-BANG (0–8)	2.86 ± 1.35	3.36 ± 1.69	0.577
Berlin questionnaire (0–10)	7.00 ± 0.00	6.10 ± 3.31	0.873
ESS (0–24)	5.33 ± 3.44	9.55 ± 4.89	0.096
SSS (0–7)	3.33 ± 1.97	3.27 ± 1.10	0.597

REM, Rapid Eye Movement; OSA, Obstructive Sleep Apnea; STOP-BANG, Snoring, Tiredness, Observed apnea, (blood) Pressure, Body Mass Index, Age, Neck Circumference, and Sex; ESS, Epworth Sleepiness Scale; SSS, Stanford Sleepiness Scale.

a Indicates a significant difference at p < 0.05. Statistically significant values are indicated in bold in the table.

## Discussion

OSA can affect the auditory system by causing chronic intermittent hypoxemia and recurrent oxygen desaturation. A higher incidence of hearing impairment in patients with OSA^11^, ^12^ or an association between tinnitus and OSA^18^, ^20^ has been previously revealed. Hearing loss is the most common cause of tinnitus; therefore, considering hearing threshold and tinnitus when analyzing their relationship with OSA is critical. Therefore, we analyzed the relationship between tinnitus and OSA in the hearing threshold-matched groups. Furthermore, most patients in this study had normal hearing thresholds to minimize the effect of hearing impairment on tinnitus.

When analyzing total sleep time, both groups demonstrated higher proportions of the N1 stage than the reference value of 5% and lower proportions of the N3 stage than the reference value of 20%.^22^ Both groups were assumed to have poor sleep quality, with longer shallow sleep times (N1) and shorter deep sleep times (N3) due to OSA. Although the N2 sleep time and percentage were statistically different between the two groups, they were not considered clinically important because the N1 and N2 values, which represent shallow sleep, were higher than the normal values in both groups.

Patients with OSA and tinnitus had a low percentage of being awake and high sleep efficiency on PSG. However, according to questionnaires, patients with OSA and tinnitus had significantly higher scores for depression (BDI-II) and daytime drowsiness (ESS). The significantly different percentages of patients who had been prescribed sedatives that affect sleep (16.7% in group 1 vs. 44.4% in group 2); (Table 1) might explain these discrepancies. We found that patients with tinnitus were prescribed medications such as clonazepam, alprazolam, pregabalin, trazodone, mirtazapine, zolpidem, and cinnarizine/dimenhydrinate, all of which affect sleep time proportions.

Previous studies reported that OSA is related to tinnitus and that OSA might be a risk factor for tinnitus due to chronic hypoxemia of the auditory system.^17^, ^23^ However, these studies were either population-based studies that reported the incidence of tinnitus and sleep disturbance or case series studies that reported the change in tinnitus before and after OSA treatment. None of them considered pure-tone thresholds between patients with and without tinnitus. Moreover, these studies did not consider both objective and subjective sleep qualities together. Others reported that tinnitus was not related to OSA in objective PSG findings but was related to poor sleep quality in sleep diary, which was similar to our findings.^18^, ^19^ However, they did not exclude the effect of hearing thresholds on tinnitus because hearing levels were normal in patients without tinnitus, whereas hearing was impaired in patients with tinnitus. Therefore, the strength of our study is that we analyzed the relationship between tinnitus and OSA in groups with similar hearing thresholds to exclude the effects of hearing loss on tinnitus. Furthermore, we analyzed objective and subjective sleep qualities using PSG and multiple sleep-related questionnaires.

The muscle tone of the upper airway decreases more, and the risk of cardiovascular complications is higher during REM sleep than during non-REM sleep; therefore, we focused on patients with REM-related OSA. Consistent with previous studies,^24^, ^25^ the proportion of women among patients with REM-related OSA was higher than that in the total patients with OSA. When analyzing the total sleep time, both groups demonstrated high proportions of the N1 stage and low proportions of the N3 stage, similar to that in the total patients with OSA; however, the difference between the two groups was absent. The patients with REM-related OSA and tinnitus had better sleep quality in the analysis of awake percentage and sleep efficiency in PSG, which is similar to the results of the total patients with OSA. Daytime drowsiness (ESS) was higher in patients with tinnitus, although the difference was statistically insignificant probably due to the small number of participants. Likewise, in all patients with OSA, the significantly different percentages of patients who had been prescribed sedatives that affect sleep (28.6% in group 1 vs. 54.4% in group 2; Table 5) might explain these discrepancies.

In summary, patients with OSA and tinnitus had better objective sleep quality (higher sleep efficiency and lower wake time) on PSG than patients without tinnitus. This unexpected result was possibly due to the effects of current medications that affect sleep induction in treating tinnitus. However, subjective sleep quality was worse in patients with OSA and tinnitus compared to those without tinnitus, possibly because chronic tinnitus could aggravate subjective sleep discomfort, which reflects long-lived patterns. These results apply to patients with both OSA and REM-OSA.

Our study is the first to identify the relationship between tinnitus and OSA or REM-related OSA in groups with similar hearing thresholds. We excluded the effect of hearing level on tinnitus by adjusting for hearing level in both groups. We evaluated objective PSG indices and subjective factors such as insomnia, anxiety, depression, daytime drowsiness, and sleep apnea using multiple questionnaires.

A limitation of our study is that since our patients’ data were retrospectively collected, we could not control for the effects of medications. In the tinnitus group, more patients were prescribed medications that affect sleep to manage their symptoms compared to the group without tinnitus. This was unavoidable due to the treatment needs of these patients. To address this limitation, we are planning a further prospective study to explore how medication usage impacts the relationship between tinnitus and sleep parameters in patients with OSA. While the observed difference in medication usage might have influenced objective sleep parameters, our results show that subjective sleep quality was poorer in the tinnitus group, even when accounting for medication effects. This suggests that chronic tinnitus itself may exacerbate subjective sleep discomfort. Another limitation is the small number of enrolled patients. Further multi-center study involving a larger population is needed to verify our results. Moreover, the relationship between severity of tinnitus and OSA parameters has not yet been investigated. Furthermore, to identify the preceding relationship between tinnitus and OSA, the changes in tinnitus after OSA treatment in the same patient groups have to be focused on in the future.

## Conclusions

We evaluated the relationship between tinnitus and OSA or REM-related OSA in groups with similar hearing thresholds. The presence of tinnitus was more strongly related to subjective sleep parameters than objective parameters. Thus, patients with OSA and tinnitus had poorer subjective sleep quality than those without tinnitus. We expect this study will contribute to gaining a deeper understanding of tinnitus and OSA. Considering the close association between tinnitus and OSA, attention should be given to tinnitus and hearing evaluation in patients with OSA.

## ORCID

Sun Choi: 0000-0003-4554-5007

Seok Hyun Park: 0000-0002-4594-5046

Su Yeon Kim: 0009-0000-8047-879X

Su Kyoung Park: 0000-0002-2274-2799

Sang Chul Park: 0000-0003-0384-8245

Jiwon Chang: 0000-0003-1660-1831

## Funding

This research was supported by Hallym University Research Fund 2021 (HURF-2021-32).

## Data statement

The authors confirm that the data supporting the findings of this study are available within the article.

## Meeting information

Some preliminary results of this study were presented as a poster at the 4th Congress of Asian Society of Sleep Medicine (ASSM) 2023 held in Bangkok, Thailand from 10th to 13th December 2023.

## Declaration of competing interest

The authors declare no conflicts of interest.
